# Supplementary material for: Polymorphic centromere locations in the pathogenic yeast Candida parapsilosis
Source: Genome Res. 2020 May;30(5):684–96. doi: 10.1101/gr.257816.119 (PMC7263194; doi:10.1101/gr.257816.119)
Supplement: Supplemental Material [file supp_30_5_684__index.html]

Polymorphic centromere locations in the pathogenic yeast Candida parapsilosis — Supplemental Material 

# Polymorphic centromere locations in the pathogenic yeast *Candida parapsilosis*

## Supplemental Material

- Supplemental\_Information.pdf
- Supplemental\_Table\_S2.xlsx
