# Supplementary material for: Polymorphic centromere locations in the pathogenic yeast Candida parapsilosis
Source: Genome Res. 2020 May;30(5):684–96. doi: 10.1101/gr.257816.119 (PMC7263194; doi:10.1101/gr.257816.119)
Supplement: Supplemental Material [file supp_gr.257816.119_Supplemental_Information.pdf]

Supplemental Information

Polymorphic centromere locations in the pathogenic yeast *Candida parapsilosis*

Mihaela Ola[1], Caoimhe E. O'Brien[1], Aisling Y. Coughlan[2], Qinxi Ma[1], Paul D. Donovan[1], Kenneth H. Wolfe[2] and Geraldine Butler[1]*.

Supplemental Figure S1. Expression of Cse4-HA and ChIP-PCR
Supplemental Figure S2. Dot matrix plots showing sequence conservation around *C. orthopsilosis* (A) and *C. metapsilosis* centromeres (B).
Supplemental Figure S3. Rearrangements at putative centromeres in *L. elongisporus*
Supplemental Figure S4. Ancestral reconstruction.
Supplemental Code. Code used to extract intergenic regions.
Supplemental Table S1. List of primers used.

Supplemental Table S2. Coordinates of centromeres in *C. parapsilosis, C. orthopsilosis* and *C. metapsilosis* (Excel file).

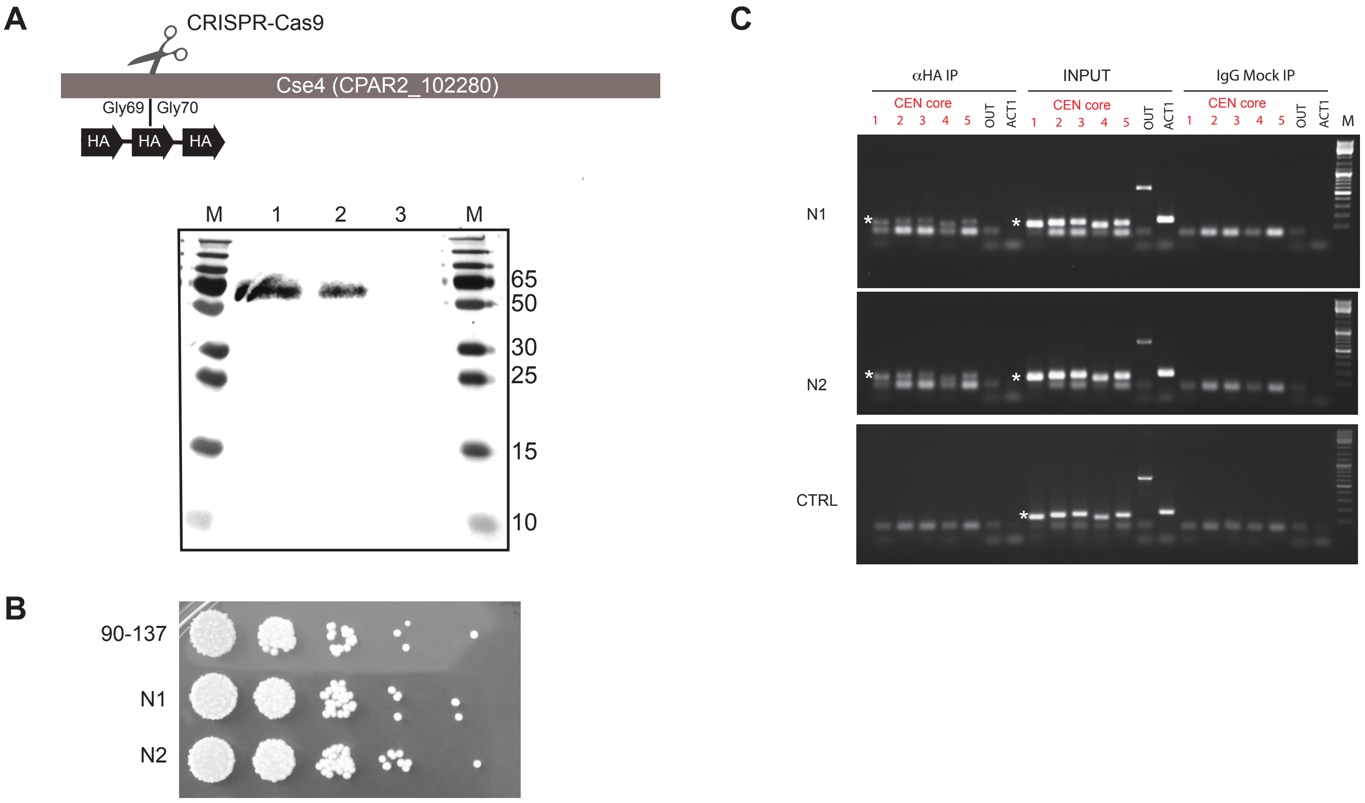

D

Repair template (modified to change PAM and add 3xHA tag)

ACCTCCCCAGTCCTTTCTCATCCCACACATGCAAGCATTGACAAATTATGGCTGATCAAC-
CAACAAATGCCACAAATCCAAGCACTACCAATGCGGCGGGTAACACCACAAGAGGTGTAG
GAGAATTAACACCTCAAGAACGCCAACTACAAGAATTGAGAAGACAAAGACAAGAACTAAG
GCGTCAACAACAACAACGGGCGCAACAACGTGCCCAACAACAAGGTTCAGCATGGGCAG
GTGCAACTGCAGGTTACCCATACGATGTTCCTGACTATGCGGGCTATCCGTATGACGTCCC
GGACTATGCAGGATCCTATCCATATGACGTTCCAGATTACGCTGGAGCAGGTTCACCAACA
CTAGCTCAATCACCGTTTGCTCGAAGAGCACAAGCAGAAGGTACAGCAACAGGCGCAATG
AGATCGCCGTTTCAGTCACCATTTGCTCGAACTAGGGATCAACAACAACAGCGACCTACAC
CTGCTCAACCAGAGAGTATACAAAGGATAAATAGAGGAGAGGGATTGGTACCACGCCCGG
GTGGTACTGGCGGAGCTGCCAGACCTTCGCCAAGGCCGGGTGCTGATATAG

Supplemental Figure S1. Expression of Cse4-HA and ChIP-PCR.

A. Expression of Cse4-HA in *C. parapsilosis* 90-137 was detected by Western blot using anti-HA antibody. Lane M: PageRuler Plus Prestained Protein ladder (Thermo Scientifics). Sizes are shown in kD. Lanes 1 and 2: protein extract from two independent Cse4-HA tagged derivatives of *C. parapsilosis* 90-137 Lane 3: protein extract from untagged *C. parapsilosis* 90-137. The protein size is expected to be 49.4 kD. **B.** Tagging Cse4 does not interfere with growth. The tag was introduced at a position that was predicted to be unlikely to interfere with the function of Cse4. Two Cse4-tagged derivatives of *C. parapsilosis* 90-137 (N1 and N2) grow as well as the parental strain *C. parapsilosis* 90-137 on YPD. Serial dilutions of cells were spotted on YPD agar and grown for 48 h at 30 degrees. **C.** ChIP-PCR from *C. parapsilosis* CEN1. N1 and N2 are Cse4-tagged derivatives, and CTRL is the untagged strain. PCR amplification was carried out using 5 pairs of primers from within the core region of CEN1 shown in red (Table S1), one pair from an adjacent intergenic region on Chromosome 1 (OUT) and one pair from ACT1. IP = HA immunoprecipitation with anti-HA; INPUT = protein extract before immunoprecipitation; mock IP = no anti-HA antibody used. The target PCR products in the core region are marked with a white asterisk. Some smaller non-specific products were also obtained. All primer pairs from within CEN1 amplified the expected size fragments from total chromatin (Input) from untagged *C. parapsilosis* 90-137 and from two Cse4-tagged strains. Some non-specific PCR products were also amplified. The CEN1-specific primers also amplified sequences from anti-HA chromatin immunoprecipitates in the tagged strains (N1 and N2), but not in the control untagged strain (CTRL). The OUT and ACT1 primers amplified products from the input samples only. Cse4 therefore localizes to the proposed CEN1. **D.** Sequence of repair template used to introduce HA tags.

A *C. orthopsilosis* centromere region

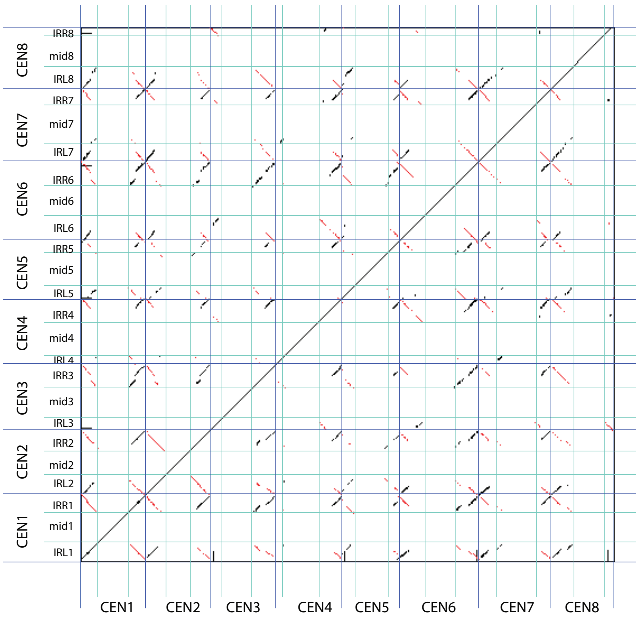

B *C. metapsilosis* centromere region

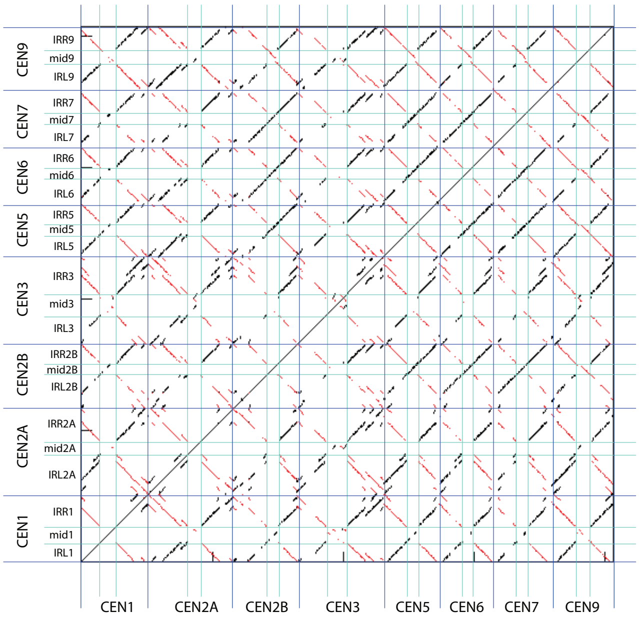

Supplemental Figure S2. Dot matrix plots showing sequence conservation around *C. orthopsilosis* (A) and *C. metapsilosis* centromeres (B). Centromeres are delineated by dark blue lines. Inverted repeats (Right, IRR and left, IRL) are separated with cyan lines. Each dot represents identity of 25-bp. Inverted sequences are shown in red, and direct repeats in black.

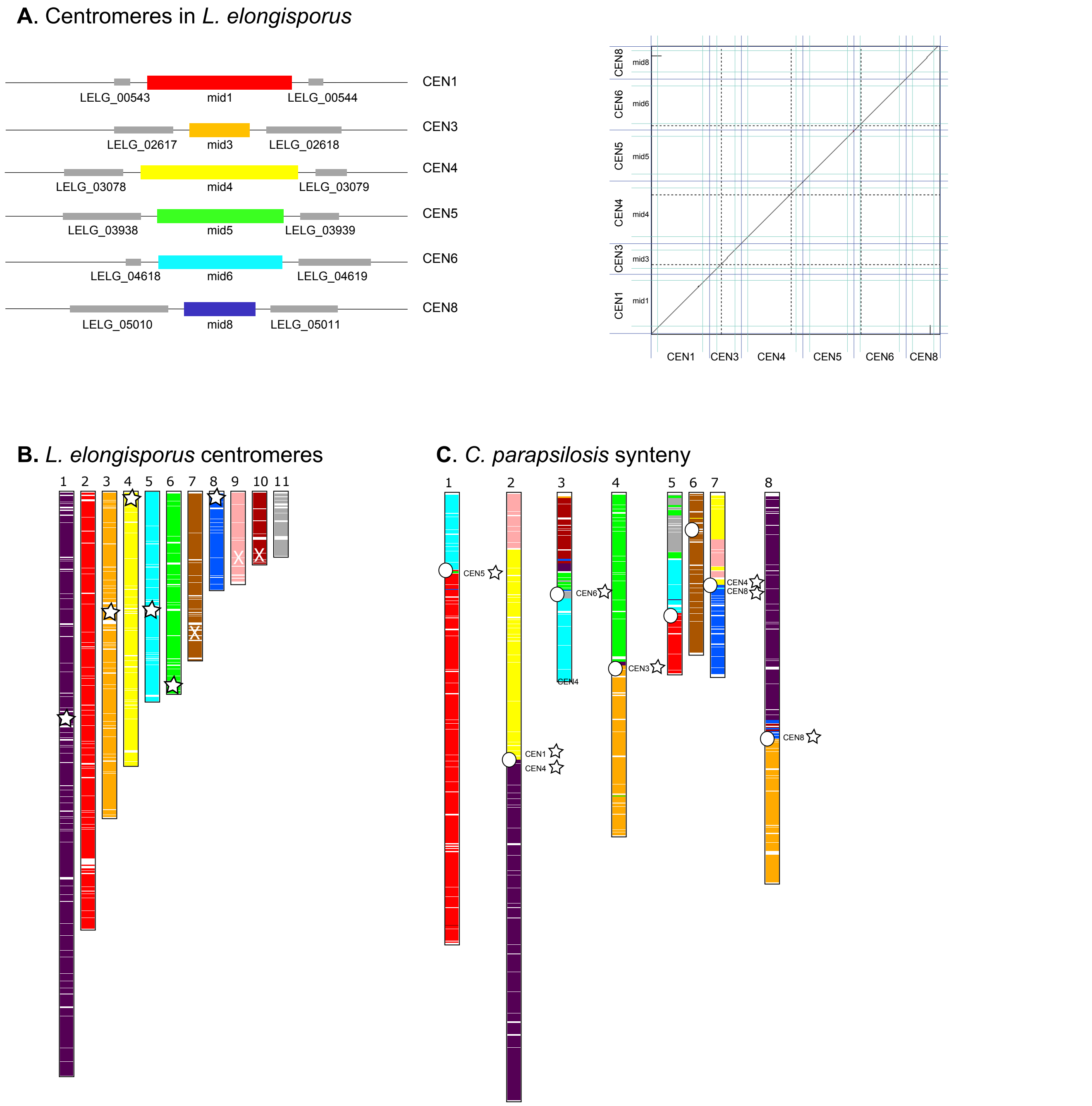

Supplemental Figure S3 Rearrangements at putative centromeres in *L. elongisporus*

A. Diagrammatic representation of centromeres in *L. elongisporus*, identified by Koren et al (2010). Only those centromeres that are likely to be correct are shown (i.e those that lie in untranscribed regions (Donovan et al 2016)). The adjacent dotplot shows that there are no repeated sequences around the centromeres.

Synteny relationship between *C. parapsilosis* and *L. elongisporus* were identified identified using SynChro.
B. Location of hits on *L. elongisporus* chromosomes. The approximate location of the putative *L. elongisporus* centromeres (from A) are indicated with white stars. Three candidates proposed by Koren et al (2010) (marked with white X's) are unlikely to represent centromeres because they are either adjacent to the rDNA (scaffold 9), or on regions that are strongly transcribed (scaffolds 7 and 10).

C. *C. parapsilosis* chromosomes, colored with respect to the RBHs from *L. elongisporus*. The location of the putative *C. parapsilosis* centromeres are indicated with a white circle. The location of syntenic *L. elongisporus* centromeres (where identified) are indicated by name and with a white star.

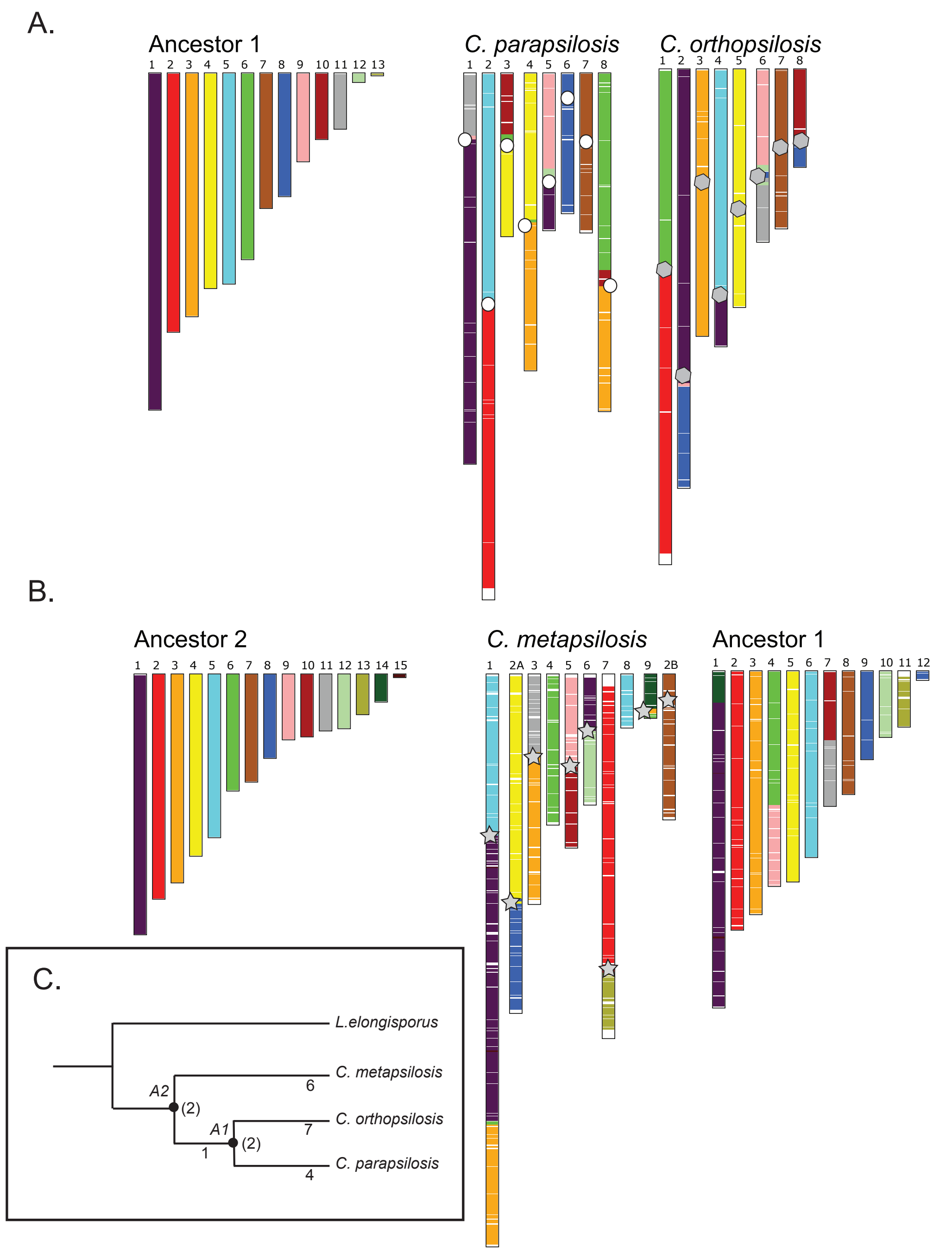

Supplemental Figure S4. Ancestral reconstruction.

A. The genome structure of the ancestor of *C. parapsilosis* and *C. orthopsilosis* (Ancestor 1, A1) was inferred using AnChro. The sytenic relationship between A1 and *C. parapsilosis* or *C. orthopsilosis* (determined using SynChro) is shown (see Fig. 4). Centromeres are indicated as in Fig. 4.

B. The genome structure of the ancestor of A1 and *C. metapsilosis* (Ancestor 2, A2) was inferred as in (A).

C. Interchromosomal breaks (ICBs) were identified by pairwise comparisons of synteny maps from SynChro. Thirteen ICBs were identified between *C. parapsilosis* and *C. orthopsilosis*. By comparing with A1, 7 were placed on the *C. orthopsilosis* branch, and 4 on the *C. parapsilosis* branch. Two (shown in parantheses) could not be placed. Similar comparisons are shown with Ancestor A2. The phylogenetic relationship between the species is taken from Pryszcz et al (2015).

Supplemental code

```
#!/usr/bin/env python3

"""
Created on Wed Apr 17 12:40:35 2019

@author: mihaela
"""

##Take in fasta file, gff file with ORF coordinates and name of first
chromosome/scaffold to be analysed
##Extracts intergenic regions and detailed information for each (i.e positions,
sequence, GC content)


import sys

fin_fasta = sys.argv[1] #input fasta file, whole genome, sequence separated by
chromosome/scaffold, given as first argument
fin_gff = sys.argv[2] #input gff with ORF coordinates, sorted by chr>>position,
given as second argument
firstChr = sys.argv[3] # first chr/scaffold name in list; given as third argument
prefix = fin_fasta[:fin_fasta.find(".")]
fout_fasta = prefix+"_chr_concat.fasta" #output fasta file with full sequences per
chromosome concatenated
fout_fasta_inter = prefix+"_intergenic.fasta" #output fasta file with sequences per
intergenic region (only the ones passing the filter)
fout_txt = prefix+"_intergenic_details.txt" #output tab delimited file with details
for each intergenic region (name, chr, start, end, sequence, length, gc-content)
filterLen = 2000 #filter for minimum size accepted

def gc_percentage(seq): #calculates the GC-content (percentage, 2 decimals)
    return round(100*(seq.upper().count("G")+seq.upper().count("C"))/len(seq),2)


def parse_fasta(file, out): #parses fasta file and creates a dictionary with chr
names as keys and sequences as corresponding fields
    sequences = {}
    sequence = ""
    name = file.readline().split()[0] #read first name
    name = name[1:]
    for line in file:
        if line[0] == ">":
            sequences[name] = sequence
            out.write("{}\t{}\n".format(name,sequence))
            name = line.split()[0]
            name = name[1:]
            sequence = ""
        else:
            sequence += line.rstrip().upper()
    sequences[name] = sequence
    return sequences


def parse_fasta_gc_content(chr_seq): #calculate GC-content for whole genome
    sequence = ""
    count = 0 #just to check it went through all the chromosome/scaffold sequences
    for chr in chr_seq.keys():
        count += 1
        sequence += chr_seq[chr].rstrip().upper()
    print("{} chromosomes/scaffolds processed for whole genome GC content.
\n".format(count))
    gc = gc_percentage(sequence)
    return gc

def parse_gff(file,chr_seq,out,inter_out): #paste gff and extract from the sequence
dictionary the intergenic regions; write them in files and save in new dictionary
for further use
```

```
    sequences = [] #list of dictionaries for all sequences
    intergenic_count = 0 #counting intergenic regions
    current_chr = firstChr #initialising first scaffold/chromosome to process
    last_position = 1 #initialising last position verified
    for line in file: # start processing gff file, line by line
        features = {} #creating one dictionary per sequence to save all details
        fields = line.rstrip().split("\t") #split gff line in fields
        # name = str(fields[3])
        chr_name = str(fields[0]) #extract chr name of current ORF
        #chr_nr = int(chr_name[-1])
        start = int(fields[3]) #extract starting position of current ORF
        end = int(fields[4]) #extract end position of current ORF
        if chr_name == current_chr: #verify if we are still on the same chromosome
            intergenic_count += 1
            features["name"] = "INTERGEN{}
{}:{}..{}".format(intergenic_count,chr_name,last_position,start-1) #create new name
for intergenic region
            features["start"] = last_position #start of intergenic region will be
last position verified, i.e. end of last ORF verified+1 or beginning of chr/scaff
            features["end"] = start-1 #end position of intergenic region will be 1
nucleotide before the current ORF
            features["chr"] = chr_name
            current_chr_seq = chr_seq[chr_name] #extract current chr sequence from
dictionary
            current_seq = current_chr_seq[last_position-1:start-1] #extract current
intergenic region sequence from the chromosome/scaffold
            features["sequence"] = current_seq
            length = len(current_seq) #length of intergenic region
            if length >= filterLen: # check if length of current intergenic region
complies to required minimum, prepare for writing, and output details
                features["length"] = length
                features["GC"] = gc_percentage(current_seq) #calculate GC content

out.write("{}\t{}\t{}\t{}\t{}\t{}\t{}\n".format(features["chr"],features["start"],f
eatures["end"],features["name"],features["length"],features["GC"],features["sequenc
e"]))

inter_out.write(">{}\n{}\n".format(features["name"],features["sequence"]))
                sequences.append(features)
            last_position = end+1

        else: #if we reached a new chromosome/scaffold, we update values and add
next intergenic region
            current_chr = chr_name
            last_position = 1
            intergenic_count += 1
            features["name"] = "INTERGEN{}
{}:{}..{}".format(intergenic_count,chr_name, last_position,start-1)
            features["start"] = last_position
            features["end"] = start-1
            features["chr"] = chr_name
            current_chr_seq = chr_seq[chr_name]
            current_seq = current_chr_seq[last_position-1:start-1]
            features["sequence"] = current_seq
            length = len(current_seq)
            if length >= filterLen:
                features["length"] = length
                features["GC"] = gc_percentage(current_seq)

out.write("{}\t{}\t{}\t{}\t{}\t{}\t{}\n".format(features["chr"],features["start"],f
eatures["end"],features["name"],features["length"],features["GC"],features["sequenc
e"]))

inter_out.write(">{}\n{}\n".format(features["name"],features["sequence"]))
                sequences.append(features)
            last_position = end+1

#open all files for reading/writting
```

```
with open(fin_fasta, "r") as fasta_in, open(fin_gff, "r") as gff_in,
open(fout_fasta, "w") as fasta_out, open(fout_txt, "w") as details_out,
open(fout_fasta_inter, "w") as inter_out:
    #create dictionary of sequences for all chr/scaffolds from input fasta
    chr_seq = parse_fasta(fasta_in,fasta_out)

    #output chromosome lengths for further construction of gff files if required
    for chr in chr_seq.keys():
        details_out.write("### {} 1..{} \n".format(chr,len(chr_seq[chr])))

    #calculate whole genome GC content if required for further comparison
    gc = parse_fasta_gc_content(fasta_in)
    details_out.write("### Overall GC content for analysed genome: {}%
\n".format(gc))
    #parse gff file and extract intergenic regions, output details in details file
and sequences in inter_out
    parse_gff(gff_in,chr_seq,details_out,inter_out)

    #close all output files
    fasta_out.close()
    details_out.close()
    inter_out.close()
```

Table S1. Oligonucleotide sequences used in this study.

Name	Sequence 5’-3’
Guide RNA construction:	
gRNA_CSE4_TOP	CCATGGGCAGGTGCAACTGCTGG
gRNA_CSE4_BOT	AACCCAGCAGTTGCACCTGCCCA
ChIP-PCR:	
CEN1	
chr1_midCEN1_1_fw	CAAGATGCCCAGAGATGCAG
chr1_midCEN1_1_rv	ATCCTACAAGTTCCTACTCG
chr1_midCEN1_2_fw	GGGATATTTCGGACAAGTAG
chr1_midCEN1_2_rv	CCAAATCAGCAACCAGCAGC
chr1_midCEN1_3_fw	GAAGAATTTCGCGTTGACTG
chr1_midCEN1_3_rv	CAAATAGTGGTCATACCGTC
chr1_midCEN1_4_fw	GCCGCCAACTTAGTTATTAC
chr1_midCEN1_4_rv	ATGAACACTTTCTCGGCATG
chr1_midCEN1_5_fw	GGATGCAGTAGTATTTGGTG
chr1_midCEN1_5_rv	CACCGTTACTGCACCCTTAC
OUT region (chr1:1948277-1955373)	
chr1_OUT_fw	TCGGCGCTAGGATCATAACA
chr1_OUT_rv	TGCCATCTTGTATTGCACCC
ACT1	
CpACT1_fw	GAAGCTTTGTTCCGTCCAGC
CpACT1_rv	TGATGGAGCCAAAGCAGTGA
Colony PCR and Repair Template (RT) amplification:	
CSE4_N_RT_fw	ACCTCCCCAGTCCTTTCTCA
CSE4_N_RT_rv	TATATCAGCACCCGGCCTTG
CSE4_col_inTag_rv	TACGGATAGCCCGCATAGTC
